# Therapeutic potential of robots for people who stutter: a preliminary study

**DOI:** 10.3389/fpsyt.2024.1298626

**Published:** 2024-01-12

**Authors:** Yuichiro Yoshikawa, Hiroaki Kobayashi, Naomi Sakai, Hiroshi Ishiguro, Hirokazu Kumazaki

**Affiliations:** ^1^Department of Systems Innovation, Graduate School of Engineering Science, Osaka University, Osaka, Japan; ^2^Department of Clinical Research on Social Recognition and Memory, Research Center for Child Mental Development, Kanazawa University, Kanazawa, Japan; ^3^Hearing and Speech Functions Section, Department of Rehabilitation for Sensory Functions, National Rehabilitation Center for Persons with Disabilities, Research Institute, Saitama, Japan; ^4^College of Science and Engineering, Kanazawa University, Kanazawa, Japan; ^5^Department of Neuropsychiatry, Graduate School of Biomedical Sciences, Nagasaki University, Nagasaki, Japan

**Keywords:** stuttering, communication robot, tandem robot, interlocutor, delayed auditory feedback

## Abstract

**Introduction:**

Growing anecdotal evidence suggests the feasibility of robotic intervention for people who suffer from disorders related to state anxiety. Few studies have been conducted on utilizing robots for persons who stutter (PWS). The present study examines the feasibility of using a robot for speech therapy for PWS.

**Methods:**

We prepared four settings (i.e., interviews with unfamiliar persons, interviews with unfamiliar communication robots, reading sentences aloud with a tandem robot that can utter the same words as a user by repeating the user’s voice after a short delay, and reading sentences aloud while being alone). We assessed the potential of the robots as both interlocutors and practice partners in training with delayed auditory feedback (DAF) for PWS. Moreover, we assessed the relationship between the trait of stuttering and the participants’ affinity to the robots.

**Results:**

Eleven PWS participated in the study. Eight (72.7%) participants had fewer stuttering-related psychological symptoms when they communicated with robots than when they communicated with humans. Spearman’s rank correlation analysis revealed that there was a significant negative correlation between the Modified Erickson Communication Attitude scale (S-24) and the difference between the scores for stuttering-related psychological symptoms pertaining to the communication robot and humans (*p* < 0.01). Six participants (54.5%) had fewer stuttering-related psychological symptoms when they read aloud with the tandem robot than when they read aloud alone. There were significant positive correlations between S-24 and the differences between the scores for stuttering-related psychological symptoms when reading aloud with the tandem robot and those when reading aloud alone (*p* < 0.01).

**Discussion:**

The communication robot and tandem utterance robot can sometimes be burdensome, although both robots were always easier to talk to for PWS in this preliminary study. The participants with positive speech-related attitudes were more inclined to decrease stuttering-related psychological symptoms when communicating with CommU than when communicating with humans. The participants whose speech-related attitudes were negative were more inclined to show a decrease in stuttering-related psychological symptoms when reading aloud with the tandem robot. Further studies are needed to provide more detailed information.

## 1 Introduction

Stuttering is a speech disorder characterized by the repetition of sounds, syllables, or words; prolongation of sounds; and interruptions in speech known as blocks. This disorder often causes difficulties in the social life of affected individuals as well as psychological distress (1).

Although stuttering is a neurodevelopmental and multifactorial disorder (2) related to state anxiety (3), its causes are not fully understood (2). The causes of stuttering have been suggested to involve genetic factors (1, 4–6) and a weak neural network in the central nervous system (5–11). Guitar suggested that the emergence and development of stuttering involve inefficient speech control and negative psychological learning (1). Inefficient speech control functions include weaknesses in language, motor, and auditory processing (12–18). Regarding psychological learning, negative reactions to stuttering, such as respondent learning or operant learning in response to reprimands or teasing from others, are believed to increase anxiety and tension related to stuttering and cause more severe symptoms, including blocking (19). Various intervention methods have been proposed for persons who stutter (PSW), including speech therapy that focuses on speech symptoms, cognitive-behavioral therapy, and dealing with stuttering-related bullying (20, 21).

Delayed auditory feedback (DAF) (16) is a well-known intervention for stuttering, and it is also known as the “echo method.” The idea behind this method is that speech disfluency disappears when PWS speak while hearing their voices over headphones with an approximate 0.2-s delay. On the other hand, frequency-altered feedback (FAF) is provided to PWS by changing the pitch of their voice (6, 22). Under FAF conditions, the voice of PWS was processed so that they hear their own voice in either a higher or lower pitch than their actual voice. Both DAF and FAF have been used to treat PWS. These methods have two effects: stimulation of auditory processing—which is underactive in PWS, enabling them to converse any way they want—and reduction in speech motor activity—which is overactive in stutterers, enabling them to perform fluency-shaping therapy outside the speech clinic (23). Although DAF and FAF have been reported to be effective for some PWS, especially those with severe stuttering (24, 25), DAF and FAF are not always effective (22).

Stuttering frequency varies according to the speech situation (2). Stuttering generally increases when speaking before many people or superiors, including teachers. However, stuttering decreases when talking to oneself, pets, stuffed toys, or in unison with others (2).

With recent rapid robotic technological advances, communication robot technology has drawn increasing attention as an effective option for the treatment or support of individuals who experience state anxiety in front of an interlocutor. Social robots can potentially engage people in an interpersonal manner by communicating and coordinating their behavior with humans through verbal, nonverbal, and/or affective modalities (26). The symptoms of PWS may be accompanied by secondary affective behaviors (e.g., avoidance behaviors and negative emotions) (27). Thus, for PWS, using robot technology in conventional interventions could introduce elements of fun, curiosity, and excitement that engage clients and make therapy experiences as well as exercises more enjoyable (28). Robots do not exhibit unconscious behaviors that could limit client engagement and comfort, and they have human-like communication and interaction, facilitating the practice of certain fluency skills (28). Robots can help adults foster social connections, combat loneliness as well as depression, and improve mood as well as quality of life (28). As per a previous study, when PWS, who have high anxiety levels, talk to a machine, their stuttering decreases (29). Further, a previous trial developed an intervention for the “echo” method using social robots for PWS (30). Given these factors and the fact that stuttering sometimes worsens when PWS experience social anxiety (31), robots may be effective for PWS who experience state anxiety in front of an interlocutor. Furthermore, repetitive practice is important for treating PWS. For example, in the integrated approach combining fluency-shaping and stuttering modification interventions, repetitive practice is performed in small steps, i.e., from short, simple utterances—including words and short sentences—to longer and more complex utterances, such as bigger sentences and conversations (1). Several studies have applied many other methods that require repetitive practice, including the Comprehensive Stuttering Program and Camperdown Program (1, 26, 32, 33).

As in other countries, Japan offers a widely used multidimensional and comprehensive approach that systematically addresses language symptoms, psychological problems, and environmental issues that plague PWS (34). Unfortunately, only a limited number of speech-language pathologists provide speech-language therapy for stuttering (35). Furthermore, only a few places exist where speech therapy can be administered, particularly for adults who stutter. Therefore, integrating robots into speech therapy to assist speech-language pathologists would be meaningful in helping PWS with limited speech therapy resources.

The primary aim of this study was to examine the potential of robotic interventions for PWS. In this study, we compared an unfamiliar person and an unfamiliar communication robot as interlocutors in the intervention. Additionally, we had the option of using another type of robot as a practice partner in the training with DAF. Therefore, we prepared four settings: interviews with unfamiliar persons, interviews with unfamiliar communication robots, reading sentences aloud while being alone, and reading sentences aloud with a tandem utterance robot that can utter the same words by repeating the user’s voice after a short delay. We sought to assess the potentiality of the robots as both interlocutors and practice partners in DAF training for PWS. Moreover, we assessed the relationship between the trait of stuttering and the participants’ affinity to the robots.

## 2 Materials and methods

### 2.1 Participants

This study was approved by the Ethics Committee of Kanazawa University. The participants were recruited through flyers explaining the details of the experiment. All procedures performed in studies involving human participants were conducted in accordance with the ethical standards of the institutional and national research committee and the 1964 Declaration of Helsinki and its subsequent amendments. After receiving a full explanation of the study, all participants agreed to participate. Written informed consent for the release of any potentially and personally identifiable images or data contained in this article was obtained from the participants. The authors declare that no conflicts of interest exist in this study.

We recruited participants who were members of self-help groups for PWS in the Kinki and Chubu regions of Japan. In this study, the inclusion criteria for the participants were (1) the diagnosis of stuttering and (2) general impact score of an Overall Assessment of the Speaker’s Experience of Stuttering for Adults (OASES) less than 1.49. Overall, 11 PWS participated in the study, but only three participants (C, E, and F) underwent speech therapy for stuttering. The existence of core stuttering speech symptoms (sound/syllable repetitions, sound prolongations, and blocks) was confirmed by an experienced speech therapist and a psychiatrist with >15 years of experience in treating PWS and social anxiety through interviews. To understand the participants’ feeling of anxiety, speech related attitude, and the impact of stuttering on a person’s quality of life, the participants completed the State–Trait Anxiety Inventory (STAI (36, 37), the Modified Erickson Communication Attitude scale (S-24) (38, 39), and the Japanese version of the OASES (40).

The STAI is a questionnaire that consists of 40 questions to be answered on a self-reporting basis and is designed to assess how strong a person’s feelings of anxiety are at a point in time, measured in relation to whether they are a natural worrier. Answers are given on a 4-point Likert scale (1—not at all, 2—somewhat, 3—moderately, 4—very much). The STAI measures two types of anxiety using subscales of 20 items each. State anxiety (anxiety about an event) assesses how the respondents feel during a stressful situation or a particular event, and trait anxiety (anxiety as a personal characteristic) evaluates how they feel in general. Higher total scores on this questionnaire indicate higher anxiety.

The S-24 is a questionnaire that has been frequently used for measuring speech-related attitudes during a variety of communication situations in adults who stutter and consists of 24 statements. The respondent indicates whether each statement is “true” or “false” in relation to his or her speech. Respondent scores are derived based on how many questions are answered as a PWS would typically respond. The total score of this test ranges from 0 to 24 points, with higher scores indicating negative speech-associated attitudes.

The OASES is a questionnaire for measuring the impact of stuttering on a person’s quality of life, including (a) general perspectives about stuttering; (b) affective, behavioral, and cognitive reactions to stuttering; (c) functional communication difficulties; and (d) the impact of stuttering on the speaker’s quality of life. Each item is rated on a 5-point scale, with higher scores indicating greater negative impact of stuttering. Impact scores can be calculated for each individual section and for all sections in total.

Each participant’s age, gender, stuttering frequency, and STAI, S-24, and OASES scores are displayed in Table 1.

**Table 1:** Demographic data of participants.

ID	Age	Sex	Stuttering frequency (%)		STAI		S-24	OASES-A-J				
			Conversation	Oral reading	State	Trait		General information	Reactions	Communication in daily life	QOL	General impact
A	26	M	11.8	20.0	40	61	18	2.94	2.77	2.45	2.92	2.76
B	30	F	1.6	2.0	46	55	18	3.11	3.33	1.96	3.48	2.98
C	32	F	0.0	2.0	28	31	8	1.95	2.00	1.36	1.48	1.7
D	68	M	2.9	0.0	43	36	9	3.00	2.40	2.08	2.12	2.36
E	38	M	3.2	0.0	50	60	8	2.44	2.77	2.46	2.92	2.67
F	28	M	0.9	0.0	45	45	19	2.70	2.41	1.68	1.72	2.11
G	27	M	2.5	0.0	29	31	14	2.19	2.47	2.38	2.16	2.32
H	20	M	8.9	10.0	35	45	19	2.38	2.67	2.78	1.95	2.46
I	19	M	14.7	20.0	47	61	18	3.13	3.97	3.58	3.25	3.54
J	23	M	38.1	64.0	44	52	18	3.15	2.67	2.70	2.58	2.75
K	36	M	1.9	0.0	40	49	8	1.84	1.87	1.57	1.40	1.67
Mean	31.6		7.86	10.73	40.64	47.82	14.27	2.62	2.67	2.27	2.36	2.48
SD	13.5		11.12	19.33	7.23	11.35	4.96	0.49	0.58	0.64	0.71	0.55

SD, standard deviation.

STAI, State–Trait Anxiety Inventory.

OASES-A-J: the Japanese version of the Overall Assessment of the Speaker’s Experience of Stuttering for Adults.

Stuttering frequency was calculated by the number of typical stuttering symptoms (repetitions, prolongations, and blocks) per 100 syllables.

### 2.2 Apparatus

#### 2.2.1 Communication robot

**Figure 1:**
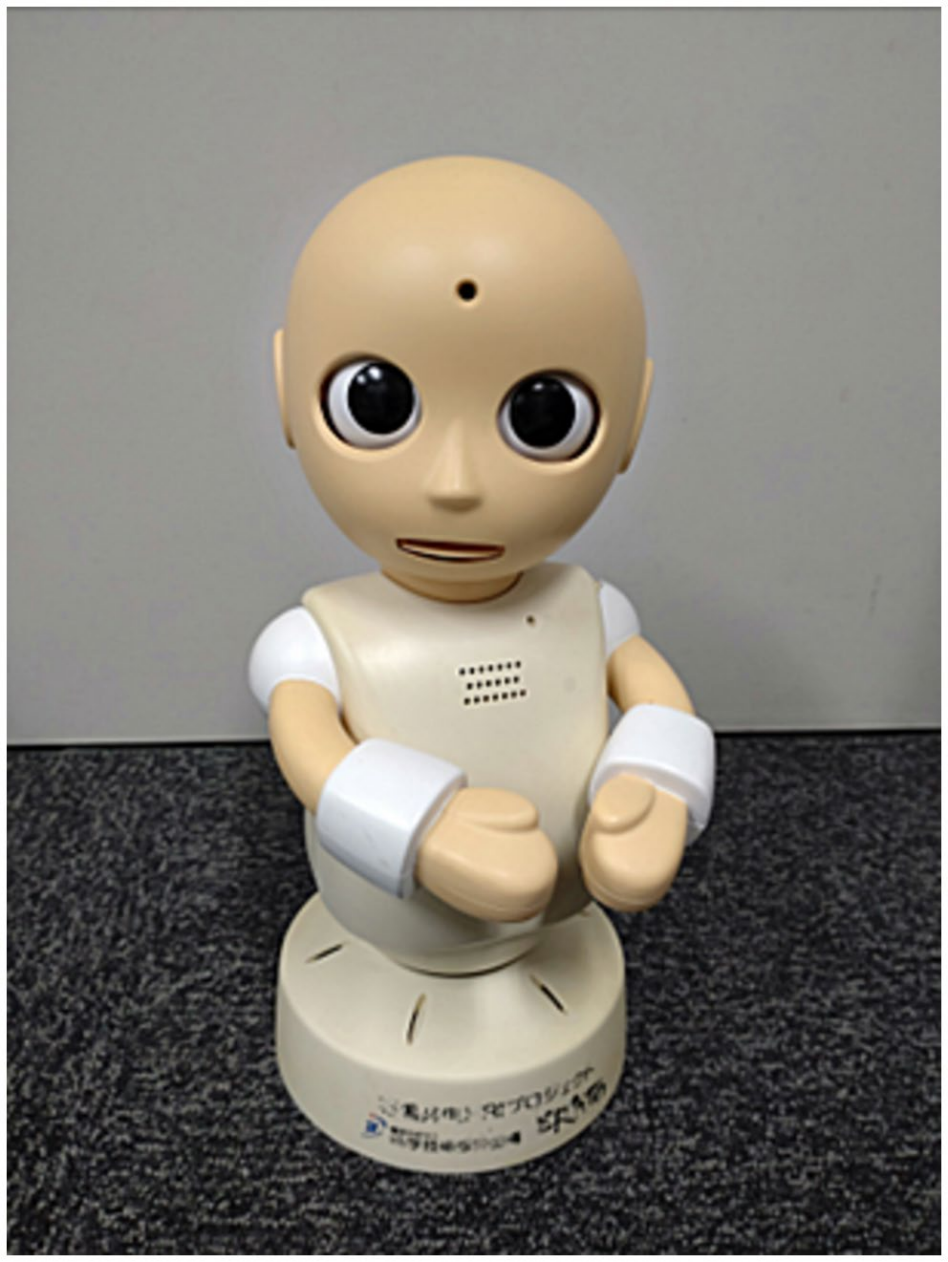
CommU.

A communication robot, CommU (Figure 1; Osaka University and Vston Co. Ltd.), with an approximate height of 30 cm, was used in this study; this robot has been used in previous protocols for individuals with ASD (41–44) but not PWS. CommU has 14 degrees of freedom (DoFs) as follows: waist (2), left shoulder (2), right shoulder (2), neck (3), eyes (3), eyelids (1), and lips (1). The careful design of the eyes and multiple DoFs dedicated to controlling its field of vision contribute to its rich gaze expressions. Its face can show a range of simplified expressions that are less complex than those of a real human face. The robot’s cute shape, which resembles a child, is expected to be easy to anthropomorphize. Furthermore, its small and cute appearance is expected to help prevent fear among individuals with PWS. In addition, CommU makes very little noise, and its controller is not distressed by its noise.

The robot was placed on the table in the experimental room and remotely controlled by web browser-based interface software. Through this software, the operator can create buttons with which they can issue commands for the robot to produce specific utterances and movements. In this experiment, the buttons for the utterances meant to be reproduced by the robot in the interview session were prepared in advance (see Supplementary file S1). An operator who monitored the experimental room pressed the appropriate button at the appropriate time so that a natural conversation between the study subject and the robot was established.

#### 2.2.2 Tandem utterance robot

**Figure 2:**
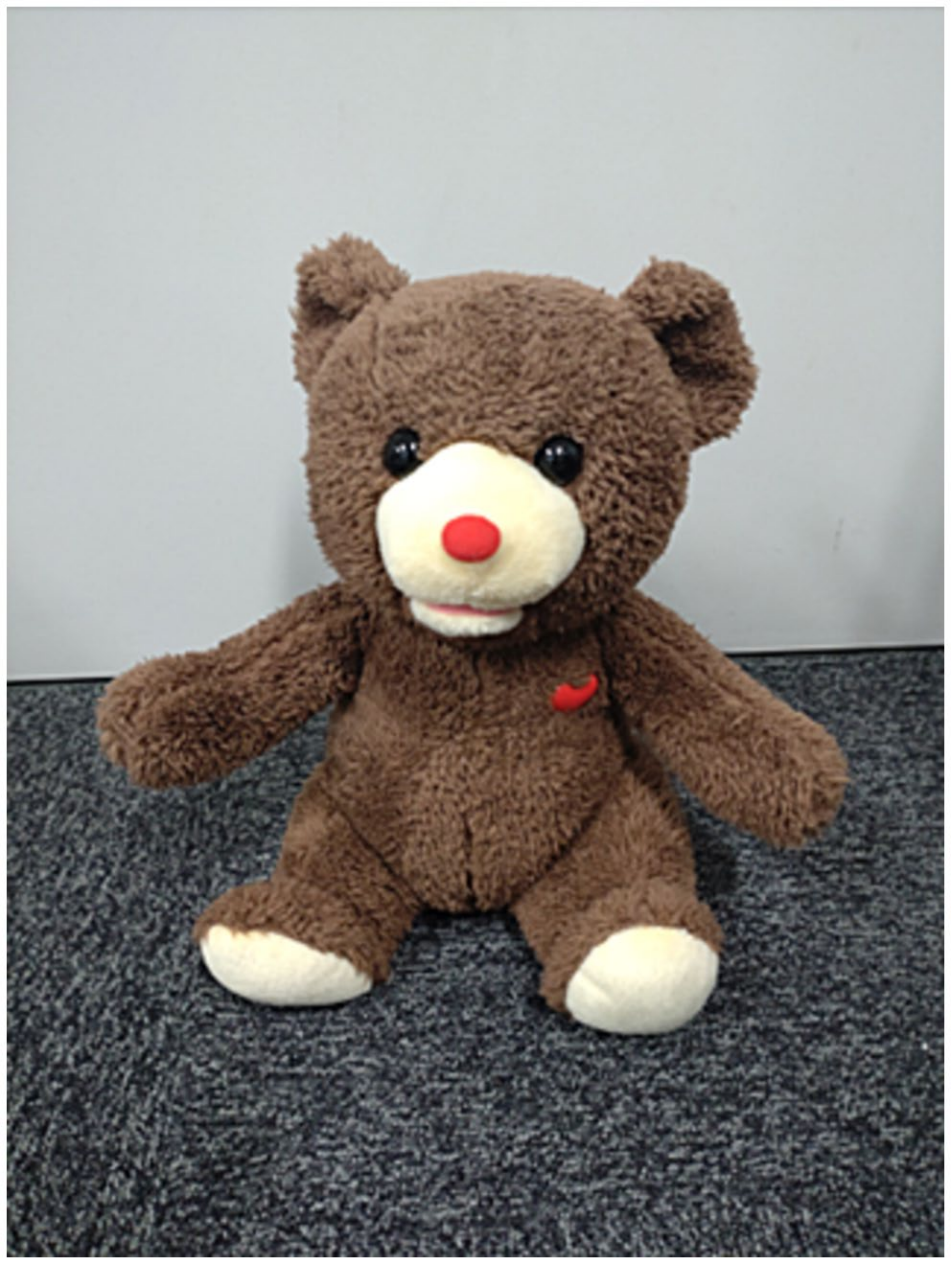
Tandem utterance robot.

A tandem utterance robot was constructed with body and voice-changing functions. For the body, a bear-looking toy robot was used, called “The Secret of the Bear” (Figure 2; T-ARTS Company, Ltd.), whose height was approximately 23 cm. This robot has been used in previous protocols for children with TD (45) but not PWS. The robot can produce an input voice from its embedded speaker with an approximate 250 ms delay while moving its mouth and waist joints along the rolling axis.

The voice of the user was exclusively collected using a small microphone array (R-Talk HS310MB, NTT Techno Cross Cooperation) and processed so that its pitch changed to approximately 400 Hz. Using this setup, when the user uttered words, the tandem utterance robot produced the same voice but delayed their voice with different pitches. Therefore, the user received both DAF and FAF. Meanwhile, the user was expected to feel like they were participating in a reading-aloud group activity with the tandem-utterance robot.

#### 2.2.3 Picture cards

In the picture card task, the participants were asked to verbally describe a picture. Two pictures were chosen from a picture set for speech therapy (46), each of which showed a scene with many people peacefully spending their time in two different situations: a family in a house and a family visiting the seaside.

### 2.3 Questionnaires

Five questions on stuttering-related psychological difficulties were formulated for this study: (1) I could speak with effort, (2) I felt anxious or tense, (3) I felt excessive tension in the throat or tongue muscles, (4) I felt anticipatory anxiety about stuttering, and (5) I noticed myself stuttering. The participants were asked to choose the most suitable option on a 5-point Likert scale (1. strongly agree, 2. moderately agree, 3. not decided, 4. moderately disagree, 5. strongly disagree). These are representative questions often used in studies on the difficulties faced by PWS (1, 47, 48).

### 2.4 Procedure

#### 2.4.1 Experiment 1: conversation with CommU or human

**Figure 3:**
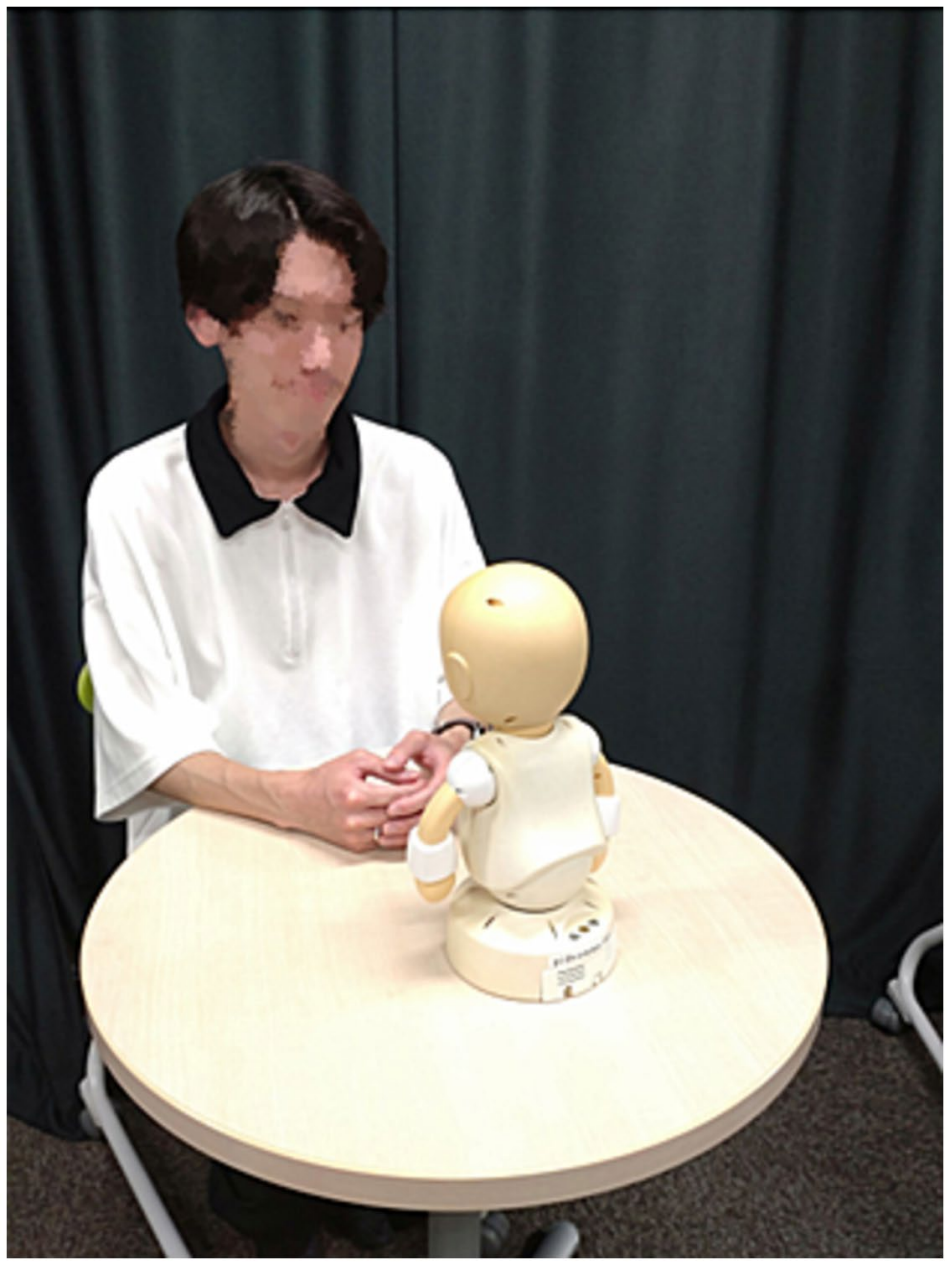
Interview session with the communication robot (i.e., CommU).

**Figure 4:**
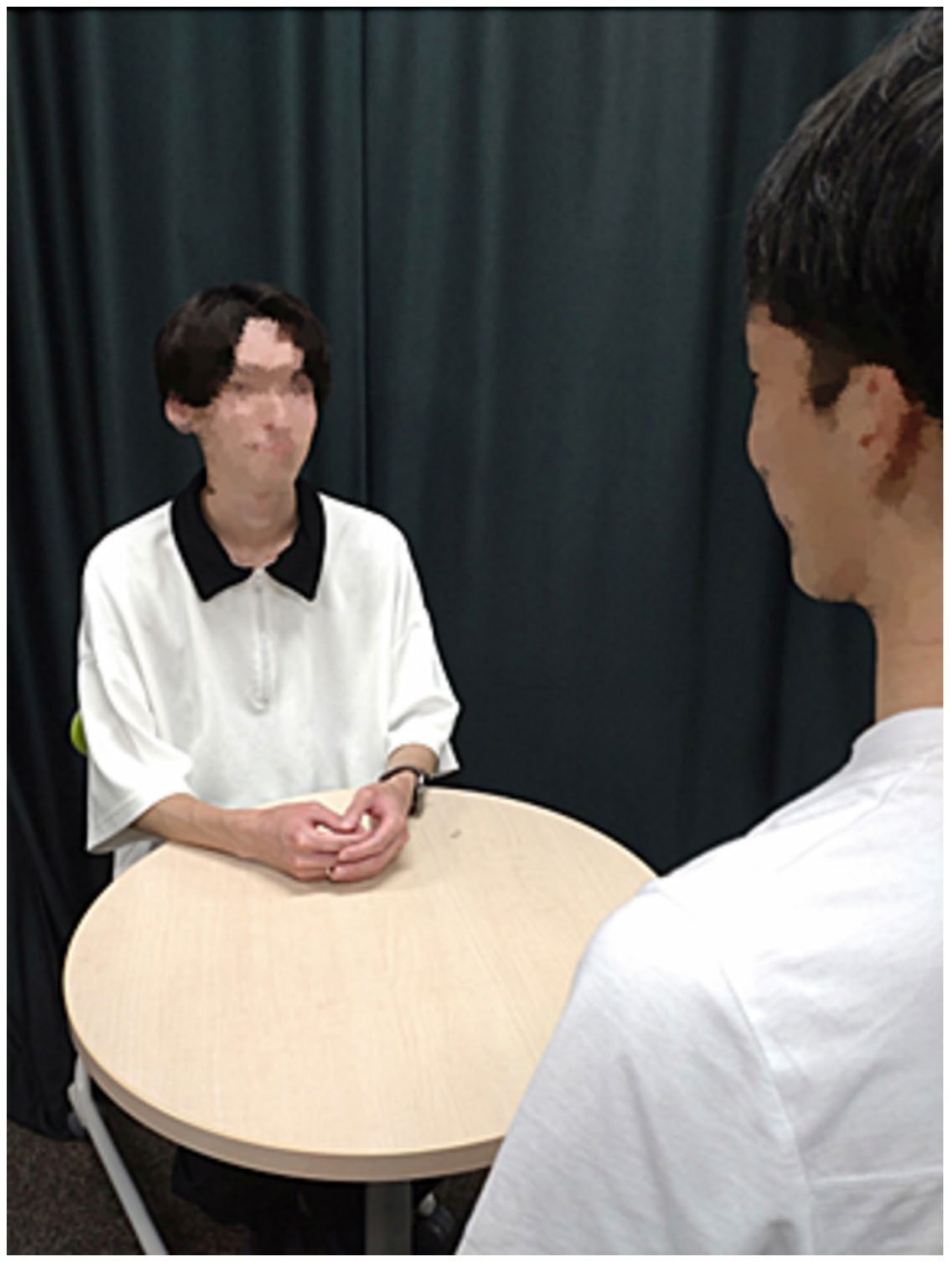
Interview session with a human.

The participants attended the first interview session with either a teleoperated communication robot (i.e., CommU) (Figure 3) or a human (i.e., experimenter) (Figure 4), during which they were asked to participate in a Q-and-A conversation and picture card task (Supplementary file S2). Each of the activities lasted approximately 2 min because we wanted to collect at least 50 utterances as the participants verbally described two pictures stated in Section 2.2.3. However, most participants uttered approximately 100 words while talking to the CommU and human in this study. The participants subsequently answered the questionnaires on stuttering-related psychological symptoms about the session. Thereafter, the participants attended the second interview session with the other interviewer, either CommU or a human, and answered the same questionnaire. The order of the interviews was randomly assigned to the participants.

#### 2.4.2 Experiment 2: reading aloud with the tandem utterance robot and reading aloud alone

**Figure 5:**
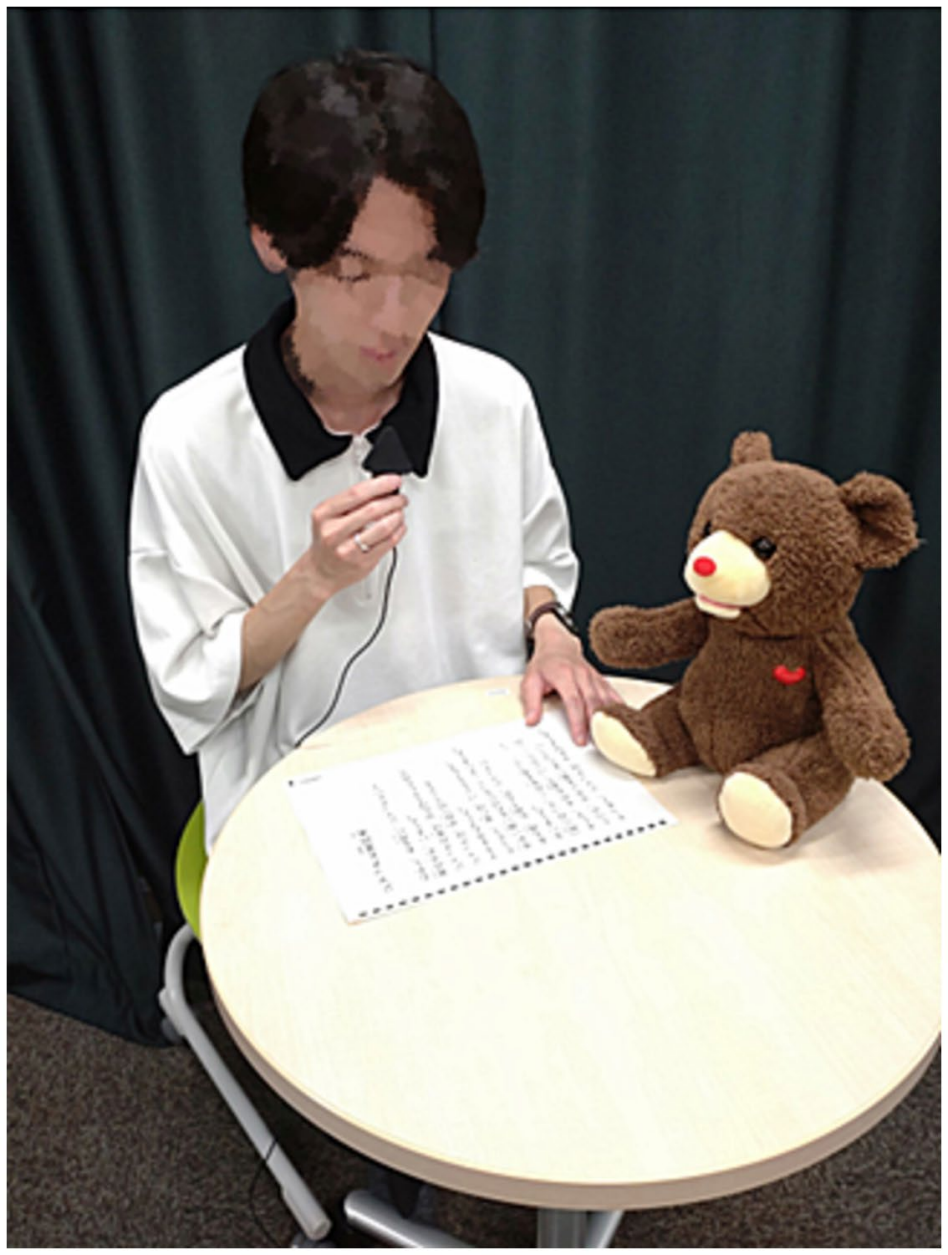
Reading aloud task with the tandem utterance robot.

**Figure 6:**
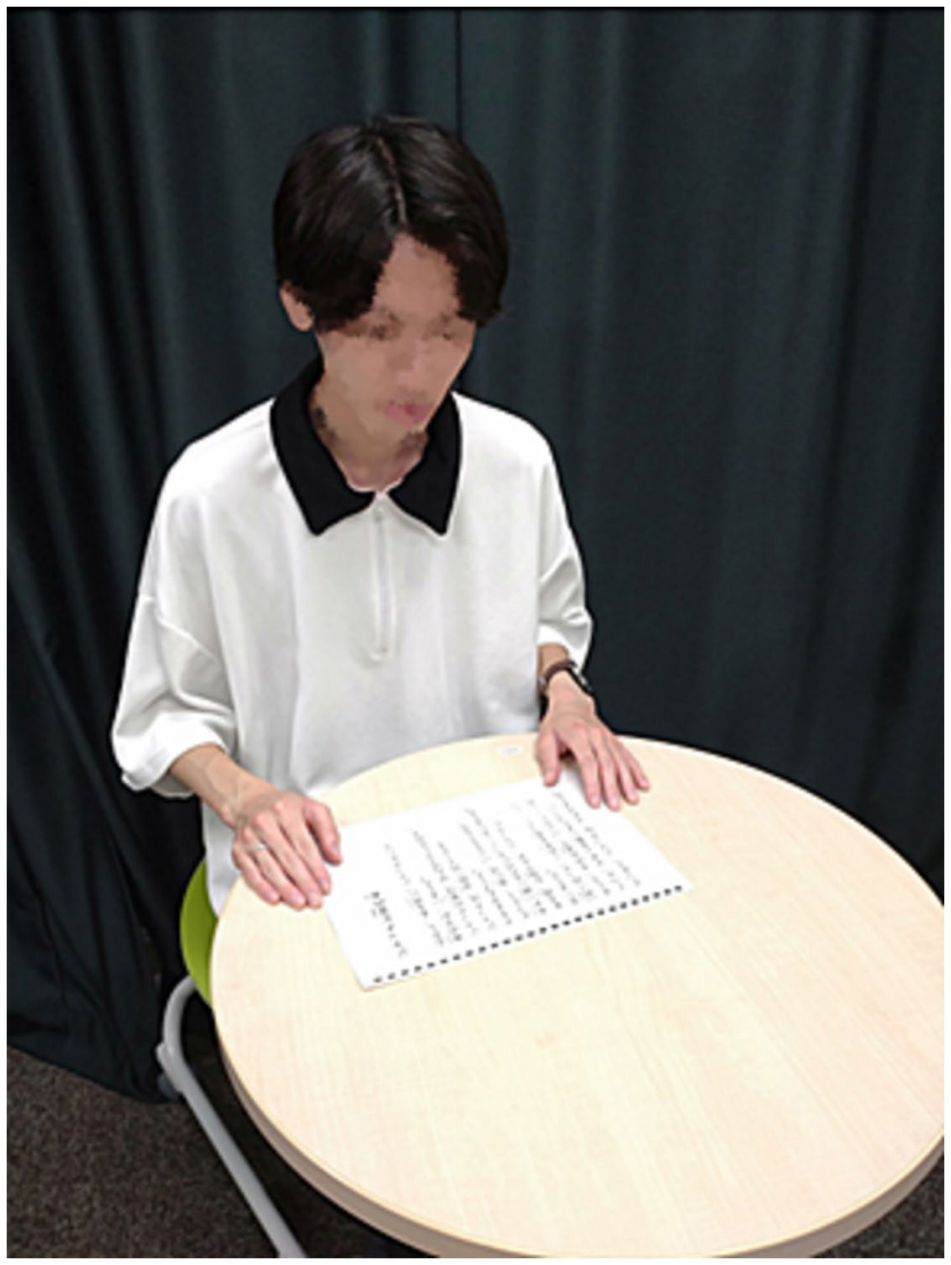
Reading aloud alone task.

Thereafter, the participants attended two sessions of the reading aloud task, each lasting approximately 2 min. Each session was conducted either with the tandem utterance robot (Figure 5) or without the tandem utterance robot (reading aloud alone) (Figure 6), followed by a questionnaire about the session. Just before the session with the robot, the participants were told how to use it and asked to utter some words to become accustomed to it. The order of the conditions was randomly assigned to each participant. The participants attending the reading aloud tasks were asked to read two sentences aloud (“Jack and the Beanstalk” and “Nature and Human”). Both reading aloud tasks consisted of 50 Japanese words that would take approximately 2 min to read. Both reading-aloud tasks were designed to meet the difficulty level of upper elementary school grades, at which typically-developing children can read without problems.

### 2.5 Statistical analysis

We performed the statistical analyses using SPSS version 24.0 (IBM, Armonk, NY, United States). The descriptive statistics of the sample were analyzed. The differences in psychological symptoms of stuttering between communication with CommU and with a human, and psychological symptoms of stuttering between communication with the tandem utterance robot and reading aloud alone were analyzed using the Wilcoxon paired rank test. We performed a Spearman’s rank correlation analysis to explore the relationships between the demographic data (i.e., stuttering frequency, STAI, S-24, OASES-A-J) and differences between the scores for stuttering-related psychological symptoms pertaining to communicating with CommU and those pertaining to communicating with humans. We also performed a Spearman’s rank correlation analysis to explore the relationships between the demographic data and differences between the scores for stuttering-related psychological symptoms pertaining to reading aloud with the tandem utterance robot or reading aloud alone. An alpha level of 0.05 was employed for these analyses.

## 3 Results

### 3.1 Experiment 1: communication with a CommU and a human

**Figure 7:**
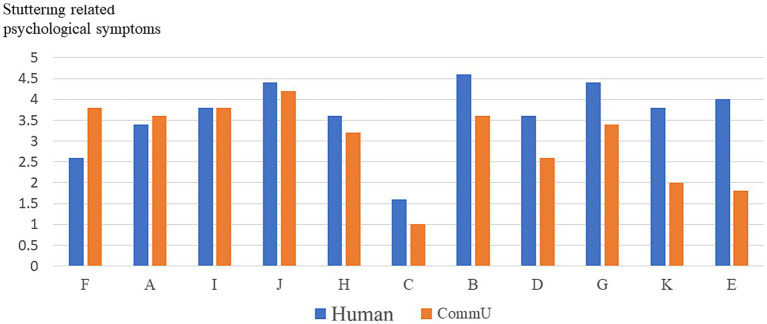
Stuttering-related psychological symptoms of all participants in communicating with CommU.

To evaluate whether communicating with CommU or with a human was easier for the participants, the differences between the stuttering-related psychological symptom scores for communicating with CommU and those for communicating with humans were calculated. If the calculated result exceeded 0, the participant could communicate with CommU more easily (that is, with fewer stuttering-related psychological symptoms) than with humans. Consequently, eight (72.7%) participants had less stuttering-related psychological symptoms when they communicated with CommU than when they communicated with humans. In contrast, other participants had the same or more severe stuttering-related psychological symptoms when they communicated with CommU. The stuttering-related psychological symptoms of all participants in each situation (i.e., communicating with CommU and humans) are shown in Figure 7.

To evaluate which situation (i.e., communicating with CommU or humans) was easier for the participants, the scores pertaining to stuttering-related psychological symptoms between CommU and human conditions were compared. There were significant differences between communicating with CommU and humans regarding the item “I could speak with effort” for stuttering-related psychological symptoms (*p* < 0.05). Details are shown in Table 2.

**Table 2:** A comparison of psychological symptoms of stuttering between communication with the CommU and human.

		CommU		Human		*p*
Item		*M*	SD	*M*	SD	
Psychological symptoms of stuttering	I could speak with effort	2.00	0.78	2.82	0.87	0.020*
	I felt anxious or increased tension	3.09	1.22	3.64	1.12	0.216
	I felt excessive tenson in my throat or tongue muscles	3.09	1.51	3.82	1.25	0.136
	I felt anticipatory anxiety of stuttering	3.18	1.47	3.64	1.29	0.271
	I noticed my own stuttering	3.64	1.12	4.18	0.87	0.237
	Total	15.00	5.06	18.09	4.35	0.055

M, mean; SD, standard deviation.

*p < 0.05.

Spearman’s rank correlation analysis revealed that there were significant negative correlations between S-24 and the difference between the scores for stuttering-related psychological symptoms pertaining to the communication robot and humans (*p* < 0.01). Details are shown in Table 3.

**Table 3:** Correlations between demographic data and differences between the scores pertaining to communicating with the CommU and those pertaining to communicating with humans.

Item	Differences between the scores pertaining to communicating with the CommU and those pertaining to communicating with humans
**Stuttering frequency**	
Conversation	−0.25
Oral reading	−0.58
**STAI**	
State	0.02
Trait	−0.19
S-24	−0.76**
**OASES-A-J**	
General information	−0.41
Reaction	−0.22
Communication	−0.24
QOL	−0.06
Overall impact	−0.27

*p < 0.05, **p < 0.01.

STAI, State–Trait Anxiety Inventory.

OASES-A-J: the Japanese version of the Overall Assessment of the Speaker’s Experience of Stuttering for Adults.

### 3.2 Experiment 2: reading aloud with the tandem utterance robot or reading aloud alone

**Figure 8:**
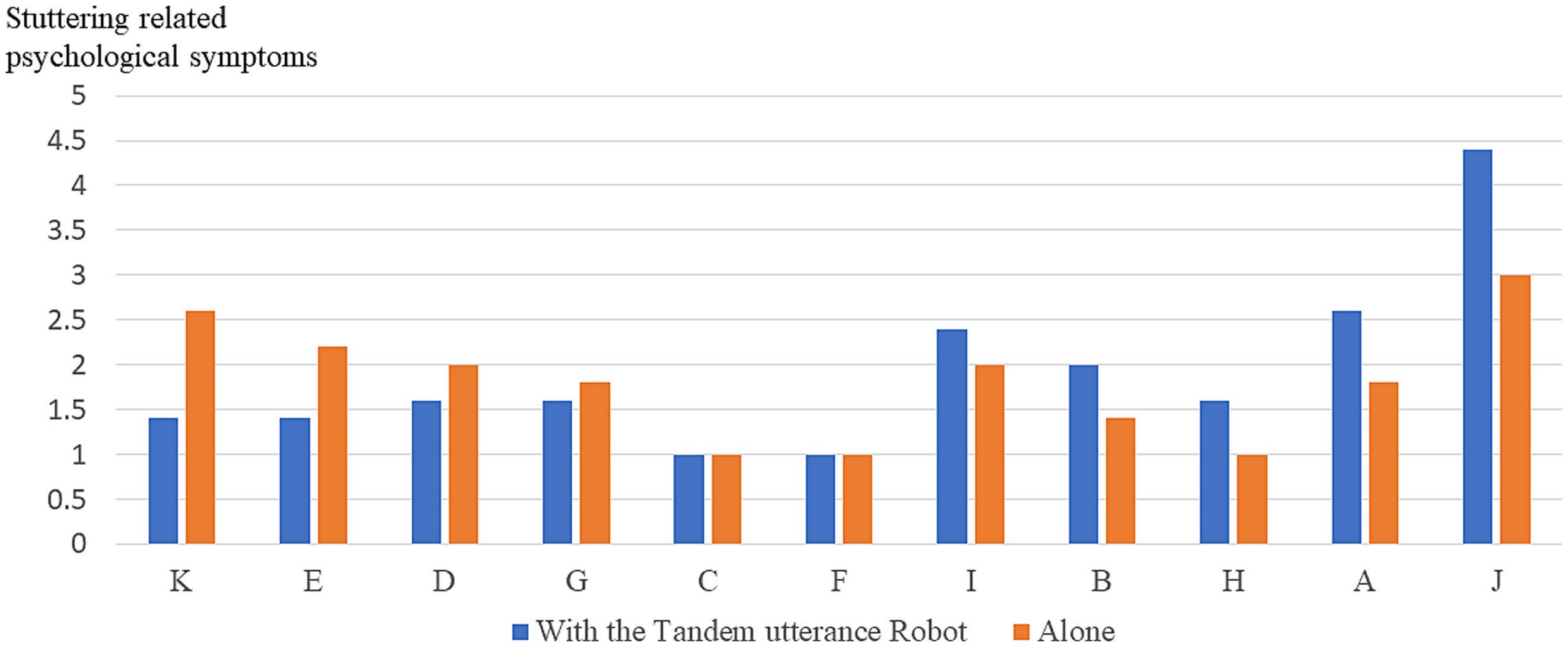
Stuttering-related psychological symptoms of all participants in communicating with a human.

To evaluate whether the reading aloud with the tandem utterance robot or reading aloud alone situations were easier for the participants, the differences between the scores of the participants reading aloud with the tandem utterance robot and those of the participants reading aloud alone were calculated. If the calculated result exceeded 0, the participant could read with the assistance of the tandem utterance robot more easily (that is, they displayed fewer stuttering-related psychological symptoms) than when reading aloud alone. Consequently, six participants (54.5%) had fewer stuttering-related psychological symptoms when they read aloud with the tandem utterance robot than when they read aloud alone. In contrast, the other participants had more stuttering-related psychological symptoms when they read aloud with the tandem utterance robot than when they read aloud alone. The stuttering-related psychological symptoms of all participants in each situation (i.e., reading alone and reading with the tandem utterance robot) are shown in Figure 8.

There were no significant differences in the stuttering-related psychological symptoms items between reading aloud with the tandem utterance robot and reading aloud alone. Details are shown in Table 4.

**Table 4:** A comparison of psychological symptoms of stuttering between reading with the tandem utterance robot and reading alone.

		Reading with the tandem utterance robot		Reading alone		*p*
		*M*	SD	*M*	SD	
Psychological symptoms of stuttering	I could speak with effort	2.82	1.47	2.82	1.17	0.652
	I felt anxious or increased tension	3.55	0.82	4.00	0.78	0.143
	I felt excessive tension in my throat or tongue muscles	2.82	1.17	3.18	1.60	0.077
	I felt anticipatory anxiety of stuttering	2.82	1.25	3.82	1.25	0.759
	I noticed my own stuttering	2.91	1.51	3.45	1.37	0.434
	Total	14.91	4.51	17.27	5.24	0.538

M, mean; SD, standard deviation.

STAI, State–Trait Anxiety Inventory.

OASES-A-J: the Japanese version of Overall Assessment of the Speaker’s Experience of Stuttering for Adults.

Spearman’s rank correlation analysis revealed that there were significant positive correlations between S-24 and the differences between the scores for stuttering-related psychological symptoms pertaining to reading aloud with the tandem robot and reading aloud alone (*p* < 0.001). Details are shown in Table 5.

**Table 5:** Correlations between demographic data and differences between the scores pertaining to reading aloud with the tandem utterance robot or reading alone.

Item	Differences between the scores pertaining to reading aloud with the tandem utterance robot or reading alone
**Stuttering frequency**	
Conversation	0.03
Oral reading	0.48
**STAI**	
State	−0.03
Trait	0.10
S-24	0.92***
**OASES-A-J**	
GI	0.33
Reaction	0.29
Communication	0.14
QOL	0.10
Overall impact	0.28

***p < 0.001.

### 3.3 Combination of the results of Experiments 1 and 2

In Experiment 1, as shown in Figure 7, for participants B, C, D, E, G, H, K, and L, communicating with CommU was easier than communicating with humans from the perspective of decreasing stuttering-related psychological symptoms. In Experiment 2, as shown in Figure 8, for participants A, B, F, H, J, and K, reading aloud with the tandem utterance robot was easier than reading aloud alone from the viewpoint of decreasing stuttering-related psychological symptoms. That is, the methods of communicating with CommU and reading aloud with the tandem utterance robot were useful for all participants in this study. Details are presented in Table 6.

**Table 6:** Differences between the scores pertaining to communicating with the CommU and those pertaining to humans and reading aloud with the tandem utterance robot and reading aloud alone.

ID	Differences between the scores pertaining to communicating with the CommU and those pertaining to humans	Differences between the scores of participants reading aloud with the tandem utterance robot and those reading aloud alone
A	−0.2	1.2
B	1	1.2
C	0.6	0.2
D	1	−0.4
E	2.2	−1.6
F	−1.2	2.4
G	1	−0.2
H	0.4	2
J	0	0.4
K	0.2	1
L	1.8	−1

## 4 Discussion

This study examined the feasibility of robotic interventions for PWS. Using CommU as an interlocutor decreased stuttering-related psychological symptoms for 72.7% of PWS in Experiment 1. Using the tandem utterance robot as a practice partner in the DAF training also decreased stuttering-related psychological symptoms for 54.5% of PWS in Experiment 2, including all PWS whose stuttering-related psychological symptoms did not decrease in Experiment 1. Thus, the results of this study suggest that both robots were effective in the treatment of the participants in this preliminary study; however, these robots can be burdensome for some participants.

In the comparison of the two conversation conditions (i.e., CommU and human), there were significant differences regarding the item “I could speak with effort” for stuttering-related psychological symptoms. This indicates that the participants conversed more effortlessly with CommU than with humans. This may suggest that talking to a robot may be easier for PWS than talking to an adult human. In general, many PWS can talk easily or more fluently when talking to themselves, dolls, pets, or young children (2). Social robots may have many advantages for PWS, including being enjoyable, engaging, and making people feel little to no judgment. Furthermore, these robots have been expected to work for PWS in the stuttering clinic by providing companionship to interlocutors (28). Therefore, it is natural that using a communication robot as an interlocutor made it easier for some PWS to converse in this study. In addition, there were significant correlations between S-24 and the differences between the scores for communicating with CommU and those for communicating with humans. This indicates that the participants with positive speech-related attitudes were more inclined to experience decreased stuttering-related psychological symptoms when communicating with CommU than when communicating with humans. For such participants, talking to CommU may have reduced their anxiety and disfluency that arise when interacting with a person.

The tandem utterance robot was easier for some PWS to talk to in Experiment 2. This may be explained by the effect of talking in unison or the DAF conditions facilitated by the tandem utterance robot. Talking in unison is efficient for decreasing stuttering speech symptoms (2). Moreover, the DAF condition is effective in decreasing stuttering speech symptoms for some PWS, especially for those with severe stuttering (7). Therefore, it is natural that the tandem utterance robot was easier to talk to for some PWS in this study. In addition, the S-24 score of the participants was positively correlated with the score difference for stuttering-related psychological symptoms between when the participants read aloud with the tandem utterance robot and when they did so alone. This finding indicates that participants whose speech-related attitudes were negative were more inclined to have decreased stuttering-related psychological symptoms when reading aloud with the tandem utterance robot. However, the mechanism between the negative attitude toward speech and the decrease in stuttering caused by the tandem robot is unclear and should be examined in future studies.

In this study, in Experiment 2, the DAF and FAF conditions were unified under one umbrella condition for all participants. Previous studies have revealed that there are extensive variations across individuals regarding the efficacious conditions of DAF and FAF (24). Therefore, for some participants, the conditions might have been ineffective because of the unification of the DAF and FAF conditions in this study. Further studies are encouraged to prepare several DAF or FAF condition sets such that every participant can select the most suitable DAF or FAF condition.

The observed decrease in stuttering-related psychological symptoms when conversing with CommU in Experiment 1 and its correlation with their speech-related attitudes (S-24) suggests that interlocutors are an important element in determining stuttering severity. The fact that stuttering-related psychological symptoms decreased for some PWS when using the tandem utterance robot having DAF and FAF functions suggests that auditory processing or speech motor activity is involved in stuttering. These findings from both Experiments (1 and 2) suggest that using robots may improve inefficient speech control functions, including the problems with auditory processing in PWS (12–18), and alleviate speech-related anxiety that results from negative psychological learning (19).

We would like to acknowledge several limitations of our study. The first is the small number of participants. In addition, the vast majority of the participants were male. Larger sample sizes and more female participants are necessary to provide more meaningful data to reveal the therapeutic potential of robots for PWS. The second limitation is the fixed choice of the interaction duration between participants and humanoid robots. A relatively short 2-min duration per session was chosen in the current experiment to avoid potential participant distress among those with high state anxiety related to long conversations. However, determining an appropriate or personalized duration for optimizing future interventions with robots is needed, where the robot’s effectiveness may be more easily and clearly evaluated. Third, importantly, our data concerning participant characteristics were based solely on self-reported measures rather than direct observation. Future studies using not only self-report measures but also behavioral observations are needed. Although this study adopted a within-subject design, participant reading difficulties may confound the results. Therefore, further investigation is required considering participant reading difficulties as potential confounding factors in robotic applications for reading aloud tasks.

This study examined the therapeutic feasibility of utilizing two types of robots for PWS. One was a small communication robot, CommU, and the other was the tandem utterance robot. Using CommU decreases stuttering-related psychological symptoms for some PWS interlocutors, as observed in Experiment 1. Using the tandem utterance robot as a practice partner in DAF and FAF training also decreased stuttering-related psychological symptoms for other PWS, as observed in Experiment 2, including all PWS whose stuttering-related psychological symptoms did not decrease in Experiment 1. These robots can sometimes be burdensome for participants with high anxiety levels, but both were easier to use in this preliminary study on PWS. Examining individual responses with TD is necessary to identify the specificity of these potential effects on PWS. As the factors influencing robot effectiveness could not be elucidated in this study, further studies are needed to provide more detailed information on this aspect; in addition, extending the use of these robots to longitudinal intervention programs to determine their effectiveness is also needed.

## Data availability statement

The original contributions presented in the study are included in the article/Supplementary material, further inquiries can be directed to the corresponding author.

## Ethics statement

The studies involving humans were approved by the Ethics Committee of Kanazawa University. The studies were conducted in accordance with the local legislation and institutional requirements. Written informed consent for participation in this study was provided by the participants’ legal guardians/next of kin. Written informed consent was obtained from the individual(s) for the publication of any potentially identifiable images or data included in this article.

## Author contributions

YY: Data curation, Formal Analysis, Methodology, Writing – original draft. HKo: Conceptualization, Data curation, Investigation, Writing – review & editing. NS: Conceptualization, Data curation, Investigation, Writing – review & editing. HI: Conceptualization, Data curation, Investigation, Writing – review & editing. HKu: Conceptualization, Data curation, Funding acquisition, Investigation, Methodology, Project administration, Writing – original draft, Writing – review & editing.
