# Supplementary material for: Therapeutic potential of robots for people who stutter: a preliminary study
Source: Front Psychiatry. 2024 Jan 12;15:1298626. doi: 10.3389/fpsyt.2024.1298626 (PMC10811234; doi:10.3389/fpsyt.2024.1298626)

Supplementary Material 1

In this experiment, buttons for the utterances of the robot to be produced in the interview session were prepared in advance as shown below.

Supplementary Figures
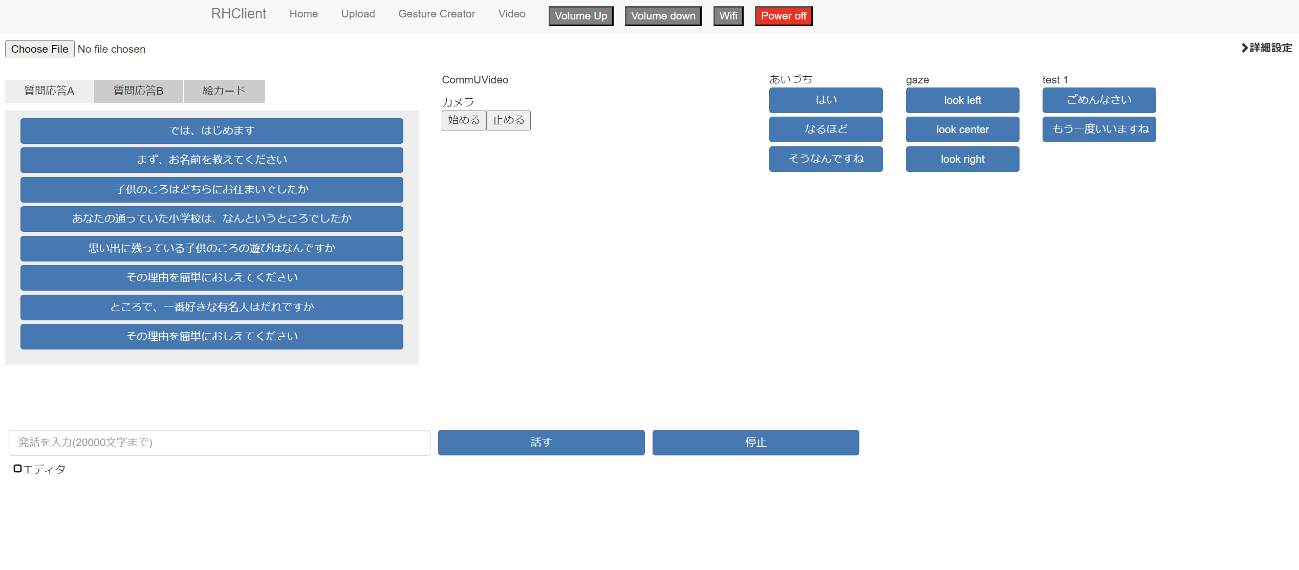

Supplement: Supplementary file 1 [file Data_Sheet_1.DOCX]
