# Supplementary material for: Therapeutic potential of robots for people who stutter: a preliminary study
Source: Front Psychiatry. 2024 Jan 12;15:1298626. doi: 10.3389/fpsyt.2024.1298626 (PMC10811234; doi:10.3389/fpsyt.2024.1298626)
Supplement: Supplementary file 2 [file Data_Sheet_2.DOCX]

Supplementary Material 2

# Example of transcripts of the Q-and-A conversation in the first session

I: Interviewer (human examinator or CommU),　P: Participant

I: “Let us begin. First, please tell me your name.”

P: (answer)

I: (utterance for acknowledgment)

I: Where did you live as a child?

P: (answer)

I: (utterance for acknowledgment)

P: (answer)

I: What was the name of the elementary school you went to?

P: (answer)

I: (utterance for acknowledgment)

I: What are some of the most memorable childhood games you played?

P: (answer)

I: (utterance for acknowledgment)

P: (answer)

I: (utterance for acknowledgment)

I: Please give me a brief reason why.

P: (answer)

I: (utterance for acknowledgment)

I: By the way, who is your favorite well-known person?

P: (answer)

I: (utterance for acknowledgment)

P: (answer)

I: (utterance for acknowledgment)

I: Please give me a brief reason why.

P: (answer)

I: (utterance for acknowledgment)

(Picture card task)

I: Please look at the picture card.

I: Please explain in detail what you see written on the picture card.

P: (answer)

I: (utterance for acknowledgment)

I: Could you please elaborate on what you just explained to me?

P: (answer)

I: (utterance for acknowledgment)

I: Please explain anything else you would like to share (if the participant gives a long explanation, this question is skipped).

P: (answer)

I: Thank you for your kind cooperation. This concludes this section.

# Example of transcripts of the Q-and-A conversation in the second session

I: Interviewer (human examinator or CommU),　P: Participant

I: Let us begin. First, please tell me your name.

P: (answer)

I: (utterance for acknowledgment)

I: Where do you live?

P: (answer)

I: (utterance for acknowledgment)

I: What is the name of the place where you were born?

P: (answer)

I: (utterance for acknowledgment)

I: What is your most memorable travel destination?

P: (answer)

I: (utterance for acknowledgment)

I: Please give me a brief reason why.

P: (answer)

I: (utterance for acknowledgment)

I: By the way, what is your favorite food?

P: (answer)

I: (utterance for acknowledgment)

I: Please give me a brief reason why.

P: (answer)

I: (utterance for acknowledgment)

# The example of scripts in the picture card task

I: Please look at the picture card.

I: Please explain in detail what you see written on the picture card.

P: (answer)

I: (utterance for acknowledgment)

I: Could you please elaborate on what you just explained to me?

P: (answer)

I: (utterance for acknowledgment)

I: Please explain anything else you would like to share (if the participant gives a long explanation, this question is skipped).

P: (answer)

I: Thank you for your kind cooperation. This concludes this section.
